# Out‐of‐field doses and neutron dose equivalents for electron beams from modern Varian and Elekta linear accelerators

**DOI:** 10.1120/jacmp.v17i4.6216

**Published:** 2016-07-08

**Authors:** Carlos E. Cardenas, Paige L. Nitsch, Rajat J. Kudchadker, Rebecca M. Howell, Stephen F. Kry

**Affiliations:** ^1^ Department of Radiation Physics The University of Texas M. D. Anderson Cancer Center Houston TX USA

**Keywords:** therapeutic electron beam, radiation leakage dose, electron applicator leakage, neutron dose equivalent, neutron source strength

## Abstract

Out‐of‐field doses from radiotherapy can cause harmful side effects or eventually lead to secondary cancers. Scattered doses outside the applicator field, neutron source strength values, and neutron dose equivalents have not been broadly investigated for high‐energy electron beams. To better understand the extent of these exposures, we measured out‐of‐field dose characteristics of electron applicators for high‐energy electron beams on two Varian 21iXs, a Varian TrueBeam, and an Elekta Versa HD operating at various energy levels. Out‐of‐field dose profiles and percent depth‐dose curves were measured in a Wellhofer water phantom using a Farmer ion chamber. Neutron dose was assessed using a combination of moderator buckets and gold activation foils placed on the treatment couch at various locations in the patient plane on both the Varian 21iX and Elekta Versa HD linear accelerators. Our findings showed that out‐of‐field electron doses were highest for the highest electron energies. These doses typically decreased with increasing distance from the field edge but showed substantial increases over some distance ranges. The Elekta linear accelerator had higher electron out‐of‐field doses than the Varian units examined, and the Elekta dose profiles exhibited a second dose peak about 20 to 30 cm from central‐axis, which was found to be higher than typical out‐of‐field doses from photon beams. Electron doses decreased sharply with depth before becoming nearly constant; the dose was found to decrease to a depth of approximately E(MeV)/4 in cm. With respect to neutron dosimetry, Q values and neutron dose equivalents increased with electron beam energy. Neutron contamination from electron beams was found to be much lower than that from photon beams. Even though the neutron dose equivalent for electron beams represented a small portion of neutron doses observed under photon beams, neutron doses from electron beams may need to be considered for special cases.

PACS number(s): 87.55.N‐, 87.55.ne, 87.56.bd, 87.56.jf

## I. INTRODUCTION

Electron beam radiotherapy is a standard treatment option in modern radiotherapy since it exhibits a sharp dose drop‐off beyond the tumor site. Unfortunately, exposure outside the target volume occurs with all radiotherapy modalities. Out‐of‐field doses have the potential to cause harm to patients and are especially problematic in pediatric or pregnant patients or those with implanted electronic devices. Furthermore, the increased survival rates over the past few decades has led to more attention on limiting dose leakage outside the target volume in order to reduce late effects and decrease the probability of secondary cancers.

For electron beam radiotherapy, out‐of‐field dose leakage is minimized by using an applicator to focus the beam and a cutout to shape the field. Dose leakage from electron therapy has been the subject of limited investigation^1^, ^2^, ^3^, ^4^, ^5^, ^6^ A better understanding of the magnitude of out‐of‐field dose associated with this treatment modality is needed, particularly for newer models of linear accelerator. The importance of studying this issue was made clear by Yeboah et al.,^(7)^ who showed very high out‐of‐field doses when utilizing electron therapy, often largely exceeding out‐of‐field doses from comparable photon treatment fields. Confounding this issue, however, is that their study was done utilizing a Siemens PRIMUS linear accelerator, which is now in limited use since it is no longer being manufactured. A comprehensive study by Alabdoaburas et al.^(8)^ reported substantially lower doses when using both Siemens ONCOR and PRIMUS linear accelerators than those observed by Yeboah and colleagues, though different electron applicators were used. In addition to secondary photon and electron doses, the production of neutrons from electron beams is virtually unexplored except for obsolete linear accelerators^(9,10)^and a single recent study.^(11)^

Given manufacturers' different approaches in designing linear accelerator heads and applicators, the apparently high variability based on these design differences, and the general lack of information on out‐of‐field doses from electron therapy and the potential risks posed by them, there is a clear need to increase the available literature on the subject. Therefore, the work presented here compares out‐of‐field doses measured for electron beams from modern Varian (Varian Associates, Palo Alto, CA) and Elekta (Elekta AB, Stockholm, Sweden) linear accelerators. This evaluation included the TrueBeam and Versa HD accelerators, which have not been examined to date. Additionally, novel neutron source strength (Q) values and neutron dose equivalents from electron beams were determined to compare values for these linear accelerators with the literature.

## II. MATERIALS AND METHODS

Doses outside of the applicator field were measured for electron beams on two Varian 21iX linear accelerators operating at 6, 9, 12, 16, and 20 MeV, a Varian TrueBeam linear accelerator operating at 6, 9, 12, 16, and 20 MeV, and an Elekta Versa HD linear accelerator operating at 6, 9, 12, and 15 MeV. The gantry and collimators were set to 0° for all measurements. The collimator jaws were set to the manufacturer's default field size (Table 1) for the energy selected. Electron dose measurements were performed using a Farmer chamber (Type 30013, PTW‐Freiburg GmbH, Freiburg, Germany) and a Wellhofer water phantom (IBA Dosimetry America, Bartlett, TN).

To determine the out‐of‐field electron dose for each combination of linear accelerator and primary electron energy used, the electron practical range, R_p_, in water was calculated from the out‐of‐field ionization depth curves measured at 10 cm from the field edge. The resultant ranges were used in calculating the most probable out‐of‐field electron energy (MeV) at the surface, $E_{p,0}$, as described by American Association of Physicists in Medicine (AAPM) Task Group ‣25: ^(12)^

(1) $$E_{p,0}(MeV)=0.22+1.98R_{p}+0.0025R_{p}{}^{2}$$

**Table 1:** X‐ray jaw opening coordinates used for each electron beam energy

	*(x,y) in cm*				
	*6 MeV*	*9 MeV*	*12 MeV*	*15/16 MeV*	*20 MeV*
Varian 21iX	(20,20)	(20,20)	(14,14)	(14,14)	(14,14)
Varian TrueBeam	(22,22)	(20,20)	(15,15)	(15,15)	(14,14)
Elekta Versa HD	(10.2,10.9)	(10,11)	(8.9,9.6)	(8.5,9.5)	

Once the most probable out‐of‐field surface electron energy was determined, the ratios of water to air mean restricted (Δ=10 ke V) collision mass stopping power values as defined by AAPM Task Group ‣25 were interpolated for each energy and utilized for dose calculation. Dose outside the treatment field $\left(D_{\text{OF}}\right)$ was calculated by

(2) DOF≈Mcorr(L¯ρ)airmedND,w60Co

where $M_{corr}$ is the fully corrected ion chamber reading, (L¯ρ)airmed is the ratio of water to air mean restricted (Δ=10 ke V) collision mass stopping powers, $N_{D,w}^{60_{Co}}$ and is the absorbed‐dose to water calibration factor for the ion chamber.

### A. Out‐of‐field dose profiles

To determine the relationship between out‐of‐field dose and distance from central‐axis, the ion chamber was placed at a depth of maximum ionization ($I_{\max}$) in water (at a source‐to‐surface distance (SSD) of 100 cm), and measurements were performed from central‐axis to a cross‐plane off‐axis distance of 40 cm from central‐axis for $10\times 10\;{\text{cm}}^{2}$ applicators. Measurements for all energies were normalized to their corresponding central‐axis dose maximum ($D_{\max}$) in the water phantom. Electron beam out‐of‐field dose profiles were compared to photon out‐of‐field dose profiles presented in the report of American Association of Physicists in Medicine Task Group ‣36.^(13)^

### B. Out‐of‐field percent depth‐dose curves

Percent depth‐dose (PDD) measurements were performed 10 cm from the field edge (15 cm from central‐axis when using $10\times 10\;{\text{cm}}^{2}$ applicator) in the cross‐plane direction by increasing ion chamber depth in water until the bremsstrahlung background was reached. The water phantom was set to 100 cm SSD. Measurements for all energies were normalized to their corresponding central‐axis dose maximum ($D_{\max}$) in the water phantom.

### C. Dose Measurements under different setup parameters

**Figure 1:**
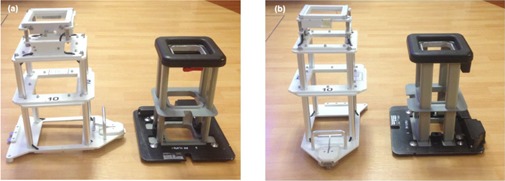
In‐plane (a) and cross‐plane (b) view of Varian 21iX and Elekta Versa HD's $10\times 10\;{\text{cm}}^{2}$ electron applicator.

The out‐of‐field dose profile and PDD measurements described above were expanded upon through the following four evaluations. These measurements were performed to provide additional information on out‐of‐field doses as a function of distance from field edge, in‐plane vs. cross‐plane direction, SSD, and applicator size. Specific details on these experiments are described below:

1. In addition to the PDD measurements at 15 cm from the central‐axis, PDD measurements were repeated on a Varian 21iX for 9 and 16 MeV electron beams. The additional PDD curves were collected at 10 and 20 cm from central‐axis (5 and 15 cm from the field edge). This experiment was performed to assess the sensitivity of PDD as a function of distance from central‐axis.
2. Because Varian 21iX applicators are not symmetric in design (see Fig. 1), out‐of‐field dose measurements were repeated on a single Varian 21iX utilizing a $10\times 10\;{\text{cm}}^{2}$ applicator in the in‐plane direction for 9 and 16 MeV electron beams. Dose profiles and PDDs were
3. collected as described in Materials & Methods sections A and B above. In‐plane doses were normalized to the in‐plane central‐axis $D_{\max}$ and compared to cross‐plane measurements from the same linear accelerator.
4. In addition to evaluating doses at 100 cm SSD, we also evaluated the effect of using an extended SSD on out‐of‐field doses. To test this effect the water phantom was set to 110 cm SSD and dose data were collected using a Varian 21iX with a $10\times 10\;{\text{cm}}^{2}$ applicator for 9 and 16 MeV electron beams. This test was performed because it has been demonstrated that utilizing extended SSD for electron beams affects the dose distribution inside the treatment field.^14^, ^15^ PDD measurements were made at a distance of 15 cm from central‐axis. For measurements made at 110 cm SSD, a water surface field size of $11\times 11\;{\text{cm}}^{2}$ was calculated; therefore, measurements at 110 cm SSD were made at 9.5 cm from field edge
5. In addition to evaluating the $10\times 10\;{\text{cm}}^{2}$ applicator, measurements were also made on a Varian 21iX utilizing a $25\times 25\;{\text{cm}}^{2}$ applicator. Dose profiles were measured at $d_{\max}$ in the cross‐plane direction. PDD curves were measured at 10 cm from field edge (22.5 cm from central‐axis). Data were collected for 9 and 16 MeV electron beams.

### D. Neutron source strength value measurements

Neutron fluence was measured on both Varian 21iX and Elekta Versa HD linear accelerators to determine neutron source strength values and neutron dose equivalents. All collimator and gantry angles were set to 0°. The neutron fluence inside the treatment room was measured using the gold‐foil activation method described in the AAPM's Report ‣19.^(16)^ To measure fast neutrons, the gold foils (∼2 cm diameter and ∼0.025 mm thick) were placed inside neutron moderators buckets (Reactor Experiments, Sunnyvale, CA). The moderators are polyethylene cylinders shielded with a boronated plastic to prevent thermal neutrons from reaching the foil. The moderators thermalize fast neutrons, which can then be captured by the foils. The induced radioactivity of the foils can then be readily measured.

**Figure 2:**
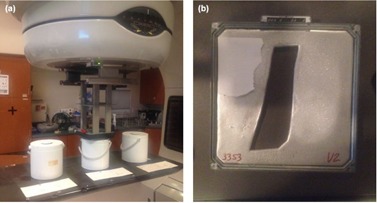
Neutron moderator buckets (a) positioned at central‐axis, 30 cm superior, and 30 cm inferior for measuring fast neutron fluence. A bare activation foil (not pictured) was positioned in the middle of the room to measure thermal neutron fluence. A $25\times 25\;{\text{cm}}^{2}$ cerrobend cutout (b) utilized to measure the increase in neutron fluence.

The moderator buckets were placed in the patient plane at central‐axis, 30 cm superior, and 30 cm inferior to central‐axis, as shown in Fig. 2(a). Gold foils were positioned perpendicular to the patient plane and were centered at a distance of 106 cm from the target on central‐axis (it was not possible to position them at 100 cm SSD because of limited clearance due to the applicator). A bare gold foil was placed in the middle of the treatment room to measure the thermal neutron fluence. Measurements were made with $10\times 10\;{\text{cm}}^{2}$ applicators on all linear accelerators, and the energies investigated were 9, 12, 16, and 20 MeV for the Varian 21iX and 9, 12, and 15 MeV for the Versa HD. Palta et al.^(17)^ concluded that neutron fluence from photon beams is influenced by collimator settings and that as collimator jaws are reduced to a $0\times 0\;{\text{cm}}^{2}$ field, neutron production increases. For electron beams, secondary collimators were set to manufacturer defaults (Table 1) to describe clinically relevant neutron fluences.

In addition to these measurements, neutron fluence measurements were made on a 20 MeV electron energy beam using a $25\times 25\;{\text{cm}}^{2}$ applicator with a large cerrobend ($Z_{\text{eff}}∼77.2$) cutout (Fig. 2(b)) in order to test neutron production in a worst‐case, clinically realistic scenario. We hypothesized that this large and highly blocked field would allow a greater number of high‐energy electrons to reach the cerrobend cutout (high‐Z material) and interact with the material to increase neutron production.

In addition to the neutron production from electron beams, neutron fluences were also measured for an 18 MV photon beam for the Varian 21iX to provide a standard comparison reference value. A collimator setting of $4\times 4\;{\text{cm}}^{2}$ was used for photon beam measurements to approximate intensity‐modulated radiotherapy settings.

Activated foils activity was counted on a calibrated Eberline model BC‐4 Beta Counter (Eberline Instrument Corp., Santa Fe, NM). The count rate per gram was established for each gold foil, and neutron fluence was calculated by using a previously established calibration factor ($3.515\times 10^{6}\;n\;{\text{cm}}^{-2}$ gram per counts $s^{-1}$). This calibration factor was determined by sending several gold foils of known mass to the National Institute of Standards and Technology to be irradiated in a known neutron fluence (±2% uncertainty) and were then counted on the Eberline counter to determine the count rate to neutron fluence relationship. The calibration factor was verified before measurements using a ${}^{90}{\text{Sr}/}^{90}Y$ source. Total fluence ($n\;{\text{cm}}^{-2}$) per unit X‐ray dose at isocenter was determined by using the method described by the National Council on Radiation Protection and Measurements (NCRP) Report 79.^(18)^ Total fluence is composed of the primary, scattered, and thermal fluence components:

(3) $$\Phi_{tot}=\Phi_{dir}+\Phi_{sc}+\Phi_{th}$$

McCall et al.^19^, ^20^ showed that the following relationship exists:

(4) $$\Phi_{tot}=\Phi_{dir}+\Phi_{sc}+\Phi_{th}=\frac{aQ}{4\pi d^{2}}+\frac{5.4aQ}{S}+\frac{1.26Q}{S}$$

where

(5) $$\begin{array}{l} \Phi_{dir}=\frac{aQ}{4\pi d^{2}}\\ \Phi_{sc}=\frac{5.4aQ}{S}\\ \Phi_{th}=\frac{1.26Q}{S} \end{array}$$

In Eqs. (4) and (5), the constant *a* is the transmission factor for neutrons that travel through the linear accelerator head shielding. This constant has a value of 0.85 for tungsten and 1.0 for lead shielding. An average value of 0.93 for the transmission factor was chosen since modern linear accelerator head shielding is manufactured employing a combination of tungsten and lead. The variable “Q” is the neutron source strength in neutrons per X‐ray dose (Gy) delivered at isocenter, the variable “S” is the surface area (cm^2^) of the treatment room, and the variable “d” is the distance (cm) from the target to the point of measurement.

Since both direct and scattered neutrons contributed to the measured fluence from the foils inside the moderator buckets, we combined direct and scattered fluences $\left(\Phi_{\text{fast}}=\Phi_{\text{dir}}+\Phi_{\text{sc}}\right)$ and solved Eq. (4) for Q:

(6) $$Q=\frac{\Phi_{fast}+\Phi_{th}}{\frac{a}{4\pi d^{2}}+\frac{5.4a}{S}+\frac{1.26}{S}}$$

For each energy evaluated, neutron source strength values were determined by averaging the results from each measurement location in the patient plane.

Neutron dose equivalent (DE) was estimated using a modified version of the McCall^(19)^method developed by Kry et al.^(21)^ McCall's formalism was modified to update the neutron radiation weighting factors and divide the fast neutron fluence into its direct and scattered components. Neutron dose equivalent was calculated using the neutron fluence and the conversion factor H:

(7) $$DE(mSv)=\frac{\Phi }{H}=\frac{\Phi_{dir}}{H_{dir}}+\frac{\Phi_{sc}}{H_{sc}}+\frac{\Phi_{th}}{H_{th}}$$

In Eq. (6), the weighting factors $H_{\text{th}}(n/{\text{cm}}^{2}/\text{mSv})=3.74\times 10^{7}$ and $H_{dir,sc}(n/cm^{2}/mSv)=1.89\times 10^{6}/{\bar{E}}_{\text{dir},\text{sc}}^{0.72}$, where $\bar{E}$ is the average neutron energy and ${\bar{E}}_{\text{sc}}=0.24\;{\bar{E}}_{\text{dir}}$ . For each linear accelerator manufacturer, we assumed the same average direct neutron energy as has been previously measured for photon therapy.^(22)^ Uncertainty in these type of measurements are typically around 20%‐30%.^21^, ^23^

## III. RESULTS

Based on Eqs. (1) and (2), ionization was converted to dose. The relevant parameters are presented in Table 2. Out‐of‐field energy values were different from in‐field energies and showed a linear relationship to in‐field values. To validate our method, out‐of‐field stopping power ratios were compared for measurements made at different distances from the field edge (as determined by the ionization depth curves measured at 10, 15, and 20 cm from central‐axis), and the stopping power ratios were found to be almost constant for all linear accelerators investigated. Uncertainties in measured electron doses were estimated to be below 5% based on repeatability of the measurements (0.5%), uncertainty on parameters such as $N_{D,w}(1\%)$ and $P_{\text{ion}}(1\%)$, and determination of the ratio of water to air mean restricted collision stopping powers (L¯ρ)airmed(2%−4%).

**Table 2:** Electron practical ranges (Rp) and probable out‐of‐field surface electron energies ($E_{p,0}$)

	*6MeV*		*9 MeV*		*12 MeV*		*15/16 MeV*		*20 MeV*	
	$R_{P}$	$E_{p,0}$	$R_{P}$	$E_{p,0}$	$R_{P}$	$E_{p,0}$	$R_{P}$	$E_{p,0}$	$R_{P}$	$E_{p,0}$
Varian 21iX ‐ 1	1.88	3.95	2.18	4.55	2.36	4.91	2.51	5.21	2.62	5.42
Varian 21iX ‐ 2	1.98	4.15	2.17	4.53	2.45	5.09	2.64	5.46	2.76	5.70
Varian TrueBeam	1.58	3.35	1.95	4.09	2.45	5.09	2.51	5.21	2.65	5.48
Elekta Versa HD	1.97	4.13	2.93	6.04	3.38	6.94	3.73	7.64		

### A. Out‐of‐field dose profiles

**Figure 3:**
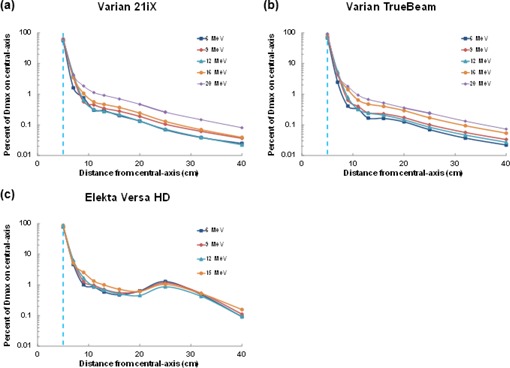
Out‐of‐field dose profiles for the (a) Varian 21iX, (b) Varian TrueBeam, and (c) Elekta Versa HD linear accelerators. Doses were normalized for each electron energy to the maximum dose value at central‐axis. The field edge is denoted by a vertical dashed blue line.

**Figure 4:**
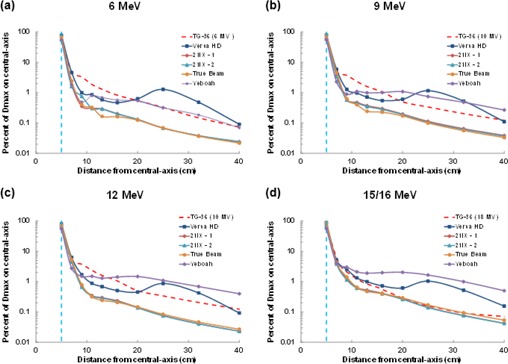
Comparison of out‐of‐field dose profiles from the linear accelerators by electron energy ((a)‐(d)), including data from Yeboah et al.^(7)^ Doses were normalized for each electron energy to the maximum dose value at central‐axis. The field edge is denoted by a vertical dashed blue line.

Figures 3 and 4 show dose profiles for a $10\times 10\;{\text{cm}}^{2}$ applicator measured from the field edge to 40 cm from central‐axis for all the linear accelerators evaluated and their respective electron energies. Figure 3 highlights electron dose as a function of treatment energy. Out‐of‐field doses were highest for the highest electron energies and typically decreased with increasing distance from the field edge. One exception was an unexpected increase in out‐of‐field dose for all Versa HD energies at a distance of 20 to 30 cm from central‐axis.

Figure 4 contrasts the out‐of‐field dose between different linear accelerators using electron beams. Substantial dose differences were observed between the Varian and Elekta linear accelerators. Negligible differences were observed between the two Varian 21iX linear accelerators where the average percent difference decreased with electron energy, from 11% for 6 MeV to 3.2% for 20 MeV. The doses from the TrueBeam were very similar to those from the 21iX linear accelerators. Out‐of‐field dose was notably higher for the Elekta Versa HD for all electron energies, particularly the low‐energy (6 and 9 MeV) electron beams.

Surprisingly, out‐of‐field doses for 12 MeV were found to be lower than 9 MeV doses for all Varian linear accelerators; this could be due to the design of the scattering foil and collimator size. The Varian and Elekta linear accelerators in this study are equipped with two scattering foils; one is used for low‐energy beams, and the other is reserved for higher (>9MeV) beams. Additionally, when increasing energy from 9 to 12 MeV, the collimator jaws decrease by 51%, 44%, and 22% in field size for the 21iX, TrueBeam, and Versa HD accelerators, respectively.

### B. Out‐of‐field percent depth‐dose curves

**Figure 5:**
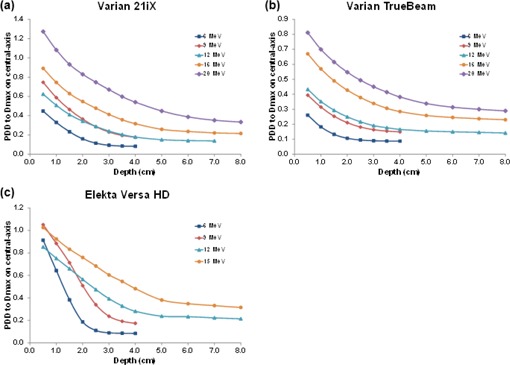
Percent depth dose (PDD) curves for the (a) Varian 21iX, (b) Varian TrueBeam, and (c) Elekta Versa HD linear accelerators. Doses were normalized for each electron energy to the maximum dose value at central‐axis.

**Figure 6:**
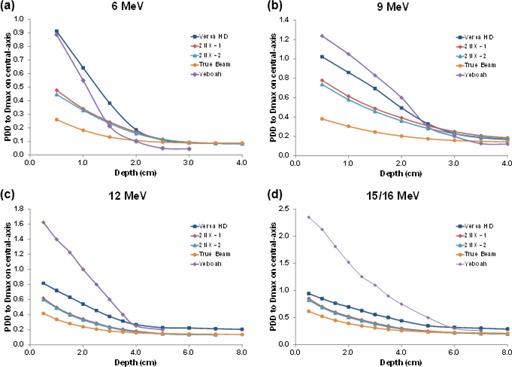
Comparison of PDD curves for linear accelerators by electron energy ((a)‐(d)). This illustration includes data from Yeboah et al.^(7)^ Doses were normalized for each electron energy to the maximum dose value at central‐axis.

Figures 5 and 6 illustrate percent depth‐dose curves measured 10 cm from the field edge utilizing a $10\times 10\;{\text{cm}}^{2}$ applicator from the linear accelerators studied and for their respective electron energies. All doses were normalized to dose maximum on the central‐axis. Figure 5 shows the PDD variation with treatment energy. Electron doses were generally higher with higher energies, except for 12 MeV beams on the Varian 21iX accelerator. On this linear accelerator, 12 MeV doses were smaller or about the same as doses from the 9 MeV beam. On the Versa HD linear accelerator, the 6 and 9 MeV beam doses decreased faster with depth than the doses from the 12 and 15 MeV beams. The scattering foil is different for the higher energy beams, which could explain the change in PDD. Importantly, without exhibiting a buildup region at shallow depths, doses decreased sharply with depth until becoming almost constant. Our results showed that the depth (cm) where the out‐of‐field bremsstrahlung background is found could be estimated by E(MeV)/4. That is, dose decreases with depth sharply until a depth of E(MeV)/4, but remains nearly constant at deeper depths.

When comparing all linear accelerators investigated (Fig. 6), the Versa HD accelerator had the highest percent depth doses for all electron energies, but when doses were reduced to their bremsstrahlung component (at relatively deep depths), the difference in dose between the linear accelerators was found to be less than 0.1% for all energies.

### C. Dose Measurements under different parameters

Results of the four evaluations to expand on out‐of‐field dose profile and PDD measurements were as follows.

**Figure 7:**
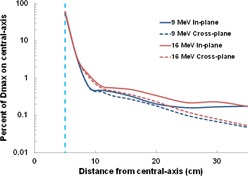
Comparison of in‐plane to cross‐plane dose profiles for 9 and 16 MeV electron beams on a Varian 21iX accelerator utilizing a $10\times 10\;{\text{cm}}^{2}$ applicator. Doses were normalized for each electron energy to the maximum dose value at central‐axis. The field edge is denoted by a vertical dashed blue line.

**Figure 8:**
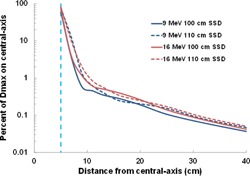
Illustration of SSD dependence on dose profiles for 9 and 16 MeV electron beams on a Varian 21iX accelerator utilizing a $10\times 10\;{\text{cm}}^{2}$ applicator. Doses were normalized for each electron energy to the maximum dose value at central‐axis. The field edge is denoted by a vertical dashed blue line.

**Figure 9:**
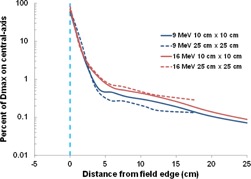
Illustration of cone size dependence on dose profiles for 9 and 16 MeV electron beams on a Varian 21iX accelerator utilizing $10\times 10\;{\text{cm}}^{2}$ and $25\times 25\;{\text{cm}}^{2}$ applicators. Doses were normalized for each electron energy to the maximum dose value at central‐axis. The field edge is denoted by a vertical dashed blue line.

1. When comparing out‐of‐field percent depth doses at different distances from the field edge, the doses were slightly higher closer to the field edge, which was expected because the dose was shown to be higher in dose profiles as measurements were made closer to field edge. However, the curve shapes remained nearly identical for all distances measured.
2. In‐plane doses and cross‐plane doses (Fig. 7) were similar for distances near the field edge, but further from central‐axis (>∼20 cm) a small but notable difference (a difference in normalized dose less than 0.15% of $D_{\max}$) was observed for both the 9 and 16 MeV beams.
3. Changing source‐to‐surface distance (Fig. 8) to 110 cm SSD resulted in a slight increase in dose profiles at both 9 and 16 MeV. For PDD measurements, our results showed slightly higher doses for 100 cm SSD (difference in PDD<0.2%) for shallow depths (<∼20 cm). The small difference in dose decreased rapidly as depth increased.
4. Changing applicator size (Fig. 9) showed a slight difference between dose profiles at distances >∼5 cm from the field edge; for the 9 MeV electron beam, doses were slightly higher when using the $10\times 10\;{\text{cm}}^{2}$ applicator, whereas for the 16 MeV beam, doses were slightly higher when using the $25\times 25\;{\text{cm}}^{2}$ applicator. This energy dependence could be attributed to the change in collimator size ($30\times 30\;{\text{cm}}^{2}$ and $28\times 28\;{\text{cm}}^{2}$ for 9 and 16 MeV, respectively, when using the $25\times 25\;{\text{cm}}^{2}$ applicator) and scattering foils, but this effect should be further investigated. These slight differences in dose found in the dose profiles translated to small dose differences in the PDD curves for both energies.

### D. Neutron source strength value measurements

Neutron source strength (Q) values determined for the Varian 21iX and Elekta Versa HD accelerators are listed in Table 3. Q values for electron beams were almost two orders of magnitude smaller than for the 18 MV photon beam for both linear accelerators. Electron beam Q values increased by a factor of 30 between 9 MeV and 20 MeV for the Varian 21iX. Q values between the Varian 21iX and the Elekta Versa HD were similar for 12 and 15/16 MeV. When the cerrobend cutout was introduced, neutron production increased by approximately 250% in comparison to 20 MeV electron beam data collected with the $10\times 10\;{\text{cm}}^{2}$ applicator. The Q value determined for the cerrobend setup was 4.8% of the Q value for the 18 MV photon beam. Even in this worst‐case scenario, the Q value for the electron beam remained at a minimum an order of magnitude lower than published data for 20 MV photon beams.^(23)^

The estimated neutron dose equivalents measured at different points are listed in Table 4. The data shows that neutron dose equivalent increased with energy and was higher on central‐axis than outside the field. Neutron dose equivalents measured at central‐axis were slightly larger for the Elekta linear accelerator, whereas out‐of‐field measurements remained about the same between both linear accelerators. Neutron dose equivalents from the 18 MV photon beam were greater than from any electron beam. Measurement on central‐axis was approximately 40 times greater for the 18 MV photon beam than for the 20 MeV electron beam, and measurements made outside the treatment field were approximately 60 times greater for the same comparison.

**Table 3:** Neutron source strength (Q) values for photon and electron beams measured on Varian 21iX and Elekta Versa linear accelerators

	*Q Value* ($x\;10^{12}$ *Neutrons per Gy)*	
	*Varian*	*Elekta*
Energy		
18 MV	1.4	
9 MeV^b^	0.0010	
12 MeV^b^	0.0032	0.0029
15/16 MeV^b^	0.015	0.018
20 MeV^b^	0.030	
20 MeV^c^	0.070	

a ^a^ Measured using $4\times 4\;{\text{cm}}^{2}$ field.

b ^b^ Measured using $10\times 10\;{\text{cm}}^{2}$ electron applicator.

c ^c^ Measured using $25\times 25\;{\text{cm}}^{2}$ electron applicator and cerrobend cutout.

**Table 4:** Neutron dose equivalents (μSv/Gy) for photon and electron beams measured on Varian 21iX and Elekta Versa HD linear accelerators utilizing a $10\times 10\;{\text{cm}}^{2}$ applicator for electron measurements

	*18 MV* ^a^	*9 MeV*	*12 MeV*		*15/16 MeV*		*20 MeV*
	*Varian*	*Varian*	*Varian*	*Elekta*	*Varian*	*Elekta*	*Varian*
Fast neutrons							
30 cm superior	1464	0.23	2.7	2.7	17	15	27
Central‐axis	2264	0.46	4.7	5.6	31	50	61
30 cm inferior	1585	0.72	3.3	2.4	12	12	27
Thermal neutrons							
Room center	31.49	0.17	0.13	0.13	0.20	0.25	0.51

a ^a^ Measured using a $4\times 4\;{\text{cm}}^{2}$ field.

## IV. DISCUSSION

In this work, we found that when using electron beams the Varian linear accelerators investigated exhibited lower out‐of‐field doses than those measured for the Elekta Versa HD. The Elekta Versa HD produced electron out‐of‐field doses that were as much as 20 times greater than the Varian linear accelerators, and at superficial depths often exceeded out‐of‐field doses associated with photon therapy based on AAPM's TG‐36 data. Out‐of‐field dose was deposited superficially, with the dose dropping rapidly to bremsstrahlung background levels within a few centimeters of the irradiated surface. Out‐of‐field doses typically increased with electron beam energy for all linear accelerators investigated, but did not change substantially with applicator size or SSD. Neutron fluences increased with electron beam energy, but were lower than the fluences associated with photon therapy.

High out‐of‐field doses have been previously noted for high‐energy electron beams. Yeboah et al.^(7)^ showed that doses outside the applicator field could be several percent (as high as 5% of central‐axis $D_{\max}$) of the given dose when utilizing the EA200 series of electron applicators on a Siemens PRIMUS linear accelerator. The measured out‐of‐field dose profiles from the Varian 21iX and TrueBeam linear accelerators were lower than the electron doses measured by Yeboah and colleagues and typical out‐of‐field dose from photon radiotherapy.^(13)^ In a more recent study, Alabdoaburas et al.^(8)^ reported lower doses (>2.5% of central‐axis $D_{\max}$) for both Siemens PRIMUS and ONCOR linear accelerators, but utilized different applicators (DEVA and Series EA3) in their measurements. The electron out‐of‐field dose profiles from both Varian linear accelerators in our study agree with the results reported by the Alabdoaburas study for the Varian 2300C/D. Notably, the electron out‐of‐field doses from the Versa HD accelerator were often higher than those seen from photon therapy (at superficial depths), and a second dose peak region was found 20 to 30 cm from central‐axis. The out‐of‐field dose from the Versa reached as high as 1.3% (at 6 MeV) of central‐axis $D_{\max}$ dose. For 6 and 9 MeV electron beams, Versa HD dose profiles were found to be higher than those in the Yeboah study in the 20 to 30 cm region, although for higher energies the study's doses with the Siemens accelerator were the highest. Both the Versa HD and Yeboah et al.'s dose profiles exhibited regions where the curves were above typical out‐of‐field doses from photon therapy reported by AAPM's Task Group ‣36. Out‐of‐field dose profiles from the Versa HD linear accelerator revealed a similar shape as the dose profiles in the PRIMUS (DEVA) and ONCOR (Series EA3); they all exhibited out‐of‐field dose peaks with the Versa HD local peak found further from central‐axis. Pitcher^(6)^found a similar second dose peak region for the Elekta Infinity linear accelerator. When using a $10\times 10\;{\text{cm}}^{2}$ applicator, cross‐plane dose profiles from the Elekta Infinity accelerator using a 7 MeV electron beam exhibited an increase in dose at a distance greater than 20 cm similar to that observed for the Elekta Versa HD accelerator. In the Pitcher study, measurements were validated by Monte Carlo simulations, which also exhibited the second dose peak region.

Out‐of‐field dose does not contribute therapeutically to the patient and it is important to minimize this unnecessary dose, especially when doses can be high. Reducing out‐of‐field dose becomes essential when treating pregnant or pediatric patients and those with implanted electronic devices. Our findings suggest that we can shield the out‐of‐field dose from electron beams by adding a water‐equivalent bolus with E(MeV)/4 thickness in centimeters. Such bolus would assure a dose less than 0.5% to a depth equal to central‐axis $d_{\max}$ for the electron energy used.

Data on neutron contamination resulting from high‐energy electron beams are limited. Followill et al.^(23)^ published a compilation of neutron source strength values for high‐energy photon beams on several linear accelerators. Our Q value for the 18 MV photon beam measured on the Varian 21iX is in agreement with the 18 MV values for Varian linear accelerators found in the literature,^22^, ^23^ whereas our Q values for the electron beams are the first to be published. Neutron dose equivalent measurements have been made by Nath et al.,^(9)^ Lin et al.,^(10)^ and Biltekin et al.^(11)^ for high‐energy electron beams. Our results are in agreement with the relatively recent findings by Lin and colleagues, who found the neutron dose equivalent from a Siemens PRIMUS linear accelerator using a 15 MeV electron beam to be approximately 20 times less than dose equivalents from a 15 MV photon beam. Biltekin et al. found neutron ambient doses using an 18 MV photon beams to be approximately 6 times greater than an 18 MeV electron beam at central‐axis; their results are found to be in agreeance with the findings of Nath et al.

## V. CONCLUSION

Out‐of‐field doses generally increased with increasing electron beam energy. When using a $10\times 10\;{\text{cm}}^{2}$ applicator, the Elekta Versa HD dose profiles exhibited a second dose peak about 20 to 30 cm from central‐axis. This second dose peak was found to be higher than even typical out‐of‐field doses from photon beams.

Dose outside the treatment field does not offer a therapeutic benefit, and requires especial attention when treating pregnant or pediatric patients and those with implanted electronic devices. Our findings suggest that adding a water‐equivalent bolus with E(MeV)/4 thickness in centimeters reduces out‐of‐field doses to less than 0.5% to a depth equal to central‐axis $d_{\max}$ for the electron energy used. Neutron contamination from electron beams was found to be much lower than that from photon beams. Even though neutron dose equivalents for electron beams represented a small proportion of neutron doses observed under photon beams, neutron doses from electron beams may need to be considered for special cases.

## ACKNOWLEDGMENTS

The authors sincerely thank the reviewers whose valuable comments have improved this paper and thank Jared Ohrt, Ramaswamy Sadagopan, Pei Wong, Scott LaNeave, Ryan Hurtt, Andrea Ohrt, Shannon Pearsall, and Luke Whittlesey for linac and equipment support. This work was supported by Public Health Service Grant CA180803 awarded by the National Cancer Institute, United States Department of Health and Human Services.

## COPYRIGHT

This work is licensed under a Creative Commons Attribution 3.0 Unported License.
